# Antimycobacterial and HIV-1 Reverse Transcriptase Activity of Julianaceae and Clusiaceae Plant Species from Mexico

**DOI:** 10.1155/2015/183036

**Published:** 2015-04-23

**Authors:** Rocio Gómez-Cansino, Clara Inés Espitia-Pinzón, María Guadalupe Campos-Lara, Silvia Laura Guzmán-Gutiérrez, Erika Segura-Salinas, Gabriela Echeverría-Valencia, Laura Torras-Claveria, Xochitl Marisol Cuevas-Figueroa, Ricardo Reyes-Chilpa

**Affiliations:** ^1^Instituto de Química, Departamento de Productos Naturales, Universidad Nacional Autónoma de México, 04510 México, DF, Mexico; ^2^Departamento de Inmunología, Instituto de Investigaciones Biomédicas, Universidad Nacional Autónoma de México, 04510 México, DF, Mexico; ^3^Hospital Infantil de México Federico Gómez, Dr. Márquez No. 162, Colonia Doctores, 06720 México, DF, Mexico; ^4^Cátedra CONACyT, Mexico; ^5^Departamento de Productos Naturales, Biología Vegetal y Edafología, Facultad de Farmacia, Universitat de Barcelona, Avenida Diagonal 643, Barcelona, 08028 Catalonia, Spain; ^6^Instituto de Botánica (IBUG), Centro Universitario de Ciencias Biológicas y Agropecuarias, Universidad de Guadalajara, 45221 Zapopan, JAL, Mexico

## Abstract

The extracts of 14 Julianaceae and 5 Clusiaceae species growing in Mexico were tested *in vitro* (50 *µ*g/mL) against *Mycobacterium tuberculosis* H37Rv and HIV reverse transcriptase (HIV-RT). The Julianaceae bark and leaf extracts inhibited *M. tuberculosis* (>84.67%) and HIV-RT (<49.89%). The Clusiaceae leaves extracts also inhibited both targets (>58.3% and >67.6%), respectively. The IC_50_ values for six selected extracts and their cytotoxicity (50 *µ*g/mL) to human macrophages were then determined. *Amphipterygium glaucum*, *A. molle*, and *A. simplicifolium* fairly inhibited *M. tuberculosis* with IC_50_ of 1.87–2.35 *µ*g/mL; but their IC_50_ against HIV-RT was 59.25–97.83 *µ*g/mL. *Calophyllum brasiliense*, *Vismia baccifera*, and *Vismia mexicana* effect on *M. tuberculosis* was noteworthy (IC_50_ 3.02–3.64 *µ*g/mL) and also inhibited RT-HIV (IC_50_ 26.24–35.17 *µ*g/mL). These 6 extracts (50 *µ*g/mL) presented low toxicity to macrophages (<23.8%). The HPLC profiles of *A. glaucum*, *A. molle*, and *A. simplicifolium* indicated that their antimycobacterial activity cannot be related to masticadienonic, 3*α*, or 3*β*-hydromasticadienonic acids, suggesting that other compounds may be responsible for the observed activity or this might be a synergy result. The anti-HIV-RT and antimycobacterial activities induced by *C. brasiliense* can be attributed to the content of calanolides A, B, as well as soulatrolide.

## 1. Introduction

Tuberculosis (TB) is an illness caused by the slow-growing acid-fast bacillus* Mycobacterium tuberculosis*. In 1993, TB declared a global emergency by the World Health Organization [1]. In 2013, there were 9 million new cases and 1.5 million deaths; this figure included 0.4 million fatalities associated with HIV patients [2].* Mycobacterium tuberculosis* is facultative intracellular bacteria that have developed resistance to first and second line antituberculosis drugs. Antibiotic resistance and multidrug-resistant TB strains are a serious problem due to the lack of results in treatment design directed to disease control and eradication [3, 4]. Due to the recent rise of TB associated with the human immunodeficiency virus VIH and the rapid spread of multidrug resistance TB strains, new classes of antimycobacterial compounds are required [5]. Compounds obtained from plants can be an important source of novel leads in the field of antituberculosis therapeutic agents [6–8], as well as against human immunodeficiency virus (HIV) [9].

Preliminary data indicate that* Amphipterygium adstringens* (Julianaceae) is a promising source of anti-TB compounds, since the stem bark extract inhibited in 95% the growth of* M. tuberculosis* at 50 *μ*g/mL; this tree species is used in Mexican Traditional Medicine for the treatment of tuberculosis and other respiratory diseases [10]. However, other 4 species of this genus found in Mexico have not been investigated yet against* M. tuberculosis *or HIV. Julianaceae species are dioecious; that is, male and female trees are found; some morphological features are useful for sex differentiation; for instance, female specimens show flowers ordinarily in groups of four in a receptacle [11]. So far, the influence of sex in the production of secondary metabolites has been poorly documented; however, in the case of* A. adstringens* bark, an accumulation of masticadienonic and 3*α*-hydroxymasticadienonic acids has been found to be higher in female plants [12].

The leaf extracts of the 23 species of Clusiaceae distributed in Mexico have been examined against HIV-1 RT [9], but not against* M. tuberculosis*. Among the 5 most active species against HIV-1 RT, the tropical tree* Calophyllum brasiliense* is remarkable [9]: its leaves contain dipirano-tetracyclic coumarins, such as calanolides A, B, and C, as well as inophyllums, mainly soulatrolide. Such compounds have been found to be active against HIV-1 RT [13] and* M. tuberculosis *[14]. The calanolide A shows potent and specific inhibition of HIV-RT [15]; this compound has been synthesized and is currently in pharmacological research phases II/III [16]. The hexane leaf extract of* C. brasiliense* has also been proposed for developing a standardized phytodrug; however, to achieve this goal, there is a need to obtain biological material with a high content of active compounds [9, 17]. The active compounds for other Clusiaceae species are still unknown.

The aim of this study was to evaluate Mexican Julianaceae and Clusiaceae crude plant extracts against* Mycobacterium tuberculosis* H37Rv and HIV-RT. Plants were selected according to two criteria: Julianaceae species, based on their use to treat tuberculosis in Mexican Traditional Medicine [18], whereas Clusiaceae species, based on bioprospective and chemotaxonomical data.

## 2. Methods

### 2.1. Plant Material

Clusiaceae and Julianaceae species were collected from different localities in Mexico (Table 1). Voucher specimens were deposited at the Herbarium Facultad de Ciencias (FCME) of the Universidad Nacional Autónoma de México and the Medicinal Herbarium (IMSSM) of Instituto Mexicano del Seguro Social.

### 2.2. Preparation of Extracts

The leaves of Clusiaceae species were used for preparing the tested extracts, whereas, in the case of Julianaceae, the extracts were prepared from the bark and leaves of specimens of different genders (male or female). All plant materials (100 g) were dried at room temperature under darkness, ground, and macerated three times for 24 h with a mixture of CH_2_Cl_2_–MeOH (1 : 1, 150 mL). The extracts were concentrated* in vacuo *to dryness and stored at room temperature until use.

### 2.3. Stock and Working Plant Extract Solution

Stock solutions of all extracts were prepared in 100% dimethyl sulfoxide (DMSO) at a concentration of 2000 *μ*g/mL and sterilized by filtration throughout a 0.22 *μ*m PTFE membrane. For* M. tuberculosis* susceptibility tests, extract solutions were prepared by diluting the stock extract in sterile 7H9 broth to obtain a 100 *μ*g/mL concentration, whereas extract solutions for anti-RT tests were diluted in the buffer provided by the kit manufacturer to obtain a working concentration of 200 *μ*g/mL (Lenti RT, Cavidi Tech).

### 2.4. Cell Culture

To assess cytotoxicity, human monocytic leukemia THP-1 cells from ATCC were cultured in RPMI 1640 medium supplemented with nonheat-inactivated 20% fetal bovine serum, 1 mM HEPES. For all experiments, THP1 were cultured in 75 cm^2^ Falcon culture flasks under standard culture conditions of 5% CO_2_ at 37°C at an initial density of 1.0 × 10^6^ cells/mL. The cultures were maintained by adding fresh medium with 10% fetal bovine serum every 2-3 days.

### 2.5. HPLC Analysis of Extracts of Julianaceae and* C. brasiliense*

**Figure 1:**
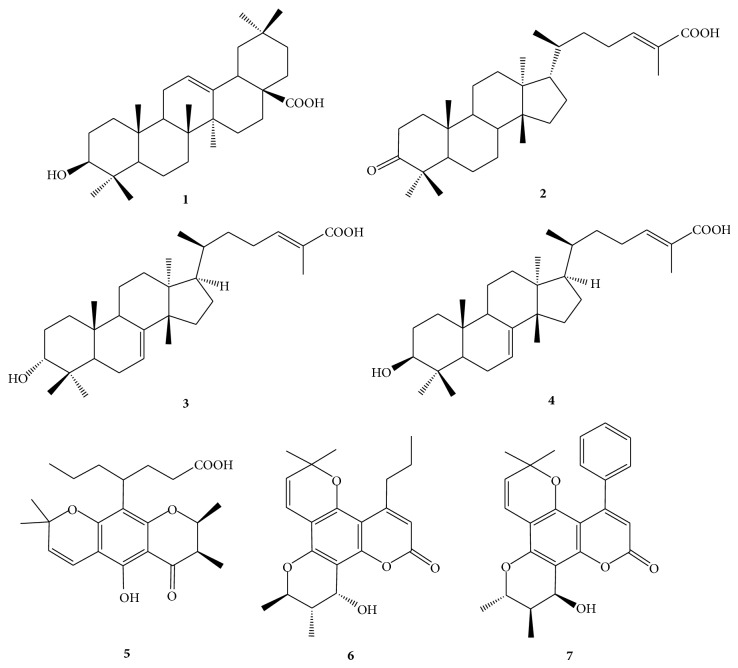
Triterpenes from Julianaceae species: oleanolic acid** 1**, masticadienonic acid** 2**, 3*α*-hydroxymasticadienonic acid** 3**, and 3*β*-hydroxymasticadienonic acid** 4**. Compounds from* C. brasiliense*: apetalic acid** 5**, calanolide B** 6**, and soulatrolide** 7**.

The bark extracts of Julianaceae and* C. brasiliense* leaves were analyzed by HPLC (Agilent 1100 series) according to previous reports [17, 19]. In the case of Julianaceae, the compounds oleanolic acid** 1**, masticadienonic acid** 2**, 3*α*-hydroxymasticadienonic acid** 3**, and 3*β*-hydroxymasticadienonic acid** 4** were quantified, whereas, for* C. brasiliense*, the concentrations of apetalic acid** 5**, calanolide B** 6**, and soulatrolide** 7** were determined (Figure 1). The chromatographic column Kromasil 100 C18, 5 *μ*m, 150 × 4.6 mm was used to analyze Julianaceae species; the mobile phase was a mixture of 0.1% aqueous acetic acid, acetonitrile containing 0.1% acetic acid and grade reagent alcohol (90% ethanol + 5% methanol + 5% 2-propanol) in a proportion 18 : 52 : 30 v/v for 25 min with an isocratic flowrate of 1.0 mL/min; the injection volume was 10 *μ*L, and the elute was analyzed at 215 nm. Each analysis was followed by a 5 min washing with 100% acetonitrile and an equilibration period with the mobile phase for 15 min.

The components of* C. brasiliense* extract were quantified using the chromatographic column Kromasil 100 C18, 5 *μ*m, 250 × 4.6 mm. The isocratic system acetonitrile water (6 : 4 v/v) with the flowrate of 1 mL/min was used for 40 min; the injection volume was 10 *μ*L and the detection wavelength 284 nm. Each analysis was followed by a 5 min washing with 100% acetonitrile, 2 min with water, and an equilibration period with the mobile phase for 3 min.

Identification of the compounds in the extracts was carried out by comparison with the retention times (RT) of pure compounds. The calibration graphs of standards were calculated and each compound was injected by triplicate over two different days; Julianaceae compounds were injected in seven different concentrations (20, 40, 60, 100, 140, 200, and 500 *μ*g/mL) whereas standards from* C. brasilense,* in six different concentrations (20, 50, 80, 120, 150, and 200 *μ*g/mL). The linear regressions and their coefficients of determination (*R* ^2^) were calculated for each compound as follows: oleanolic acid** 1**, *y* = 3.7085*x* − 17.043, 0.9987; masticadienonic acid** 2**, *y* = 10.766*x* + 4.3811, 0.9990; mixture of 3*α* and *β*-hydroxymasticadienonic acids (3 & 4), *y* = 11.466*x* + 22.14, 0.9993; apetalic acid** 5**, *y* = 19.547*x* + 135.64, 0.9933; calanolide B** 6**, *y* = 27.786*x* + 13.369, 0.9995 and soulatrolide** 7**, *y* = 35.075*x* + 209.85, 0.9934. Finally, the percentage of each compound in the extracts was calculated interpolating the linear regression equation. The results are reported as the percentage of extract (Table 3).

### 2.6. HIV-1 RT Inhibition Test

The extracts were evaluated by a nonradioactive immunocolorimetric assay (Lenty RT Activity Assay, Cavidi Tech) according to the protocol provided by the manufacturer. All extracts were first tested at 50 *μ*g/mL with a final DMSO concentration of 0.5% v/v. Reported values are means of 5 replicates ± SEM. The IC_50_ values were calculated only for extracts that inhibited ≥50% the enzymatic activity. These extracts were tested at 7 concentrations 3.125 to 200 *μ*g/mL with increments of 0.3 logarithms. Reported values are means of 3 replicates ± SEM. Nevirapine, a nonnucleoside reverse transcriptase inhibitor (NNRTI), was used as a positive control from 0.01 *μ*M to 1 mM with increments of 1 logarithm.

### 2.7. Antimycobacterial Screening by Microplate Alamar Blue

The activity of all extracts was tested using the microplate Alamar blue assay as previously described [20, 21]. Outer wells were filled with sterile distilled water (200 *μ*L) to prevent dehydration in experimental wells. Colum 2 (B to G wells) was used to evaluate the reference drug rifampin; serial twofold dilutions in 100 *μ*L of Middlebrook 7H9 medium were performed to obtain concentrations from 2.0 to 0.06 *μ*g/mL. Wells 10 E and F were used for DMSO control, and wells 11 B to 11 E for the drug free control. One-hundred *μ*L of supplemented 7H9 broth plus 100 *μ*L the bacterial inoculum (1 × 10^6^ ufc/mL) was added to each of these wells. Simultaneously a diluted control 1 : 100 was prepared from the bacterial suspension, representing 1% growth of the bacterial population tested. All other wells received 100 *μ*L of the extract solution (100 *μ*g/mL) and 100 *μ*L bacterial inoculums. The final concentration of DMSO in well was <1.0% v/v, and all extracts were tested at 50 *μ*g/mL. The IC_50_ values were calculated only for those extracts that inhibited ≥50% the mycobacterial growth; these extracts were tested at seven concentrations (3.125 to 200 *μ*g/mL) with increments of 0.3 logarithms. Each microplate was incubated for 7–10 days at 37°C; after incubation, one growth control was developed with a mixture of 20 *μ*L of Alamar blue solution (ABD Serotec) and 5 *μ*L of sterile 20% Tween 80. The plates were reincubated at 37°C for 24 h. After this period, if the control well turned from blue (no growth) to pink (growth), the remaining wells were treated with Alamar-Tween, as previously described, and incubated for additional 24 h. Reduction of Alamar blue was calculated according to the manufacturer protocol. Optical density of the plate was measured at 540 and 600 nm with a spectrophotometer. The percentage of inhibition of the crude extracts was defined as 100 − percentage of reduction of Alamar blue.

### 2.8. Cytotoxicity Assay

Crude extracts were evaluated against human macrophages THP1 cell line. The differentiation of THP1 cells was performed with PMA (phorbol 12-myristate 13 acetate) 50 nM [22]. Twenty thousand cells in the differentiation process were placed in each well, and the plates were incubated for 72 h at 37°C, and 5% CO_2_ atmosphere. After the incubation, the plates were washed twice with RPMI supplemented medium and 100 *μ*L of the extract solutions (50 *μ*g/mL) was added to each well and reincubated for 24 h. After the reincubation time 10 *μ*L of Alamar blue solution was added to each well, and the plates were reincubated for 24 h. The anthracycline doxorubicin was used as a positive control, and the data were interpreted as indicated by the manufacturer. Cytotoxicity was calculated as the ratio of the average OD (570 and 600 nm) obtained as compared with control wells (untreated macrophages).

## 3. Results

### 3.1. Screening of Plant Extracts

The 14 Julianaceae extracts displayed high antimycobacterial activity (>84%). These results are consistent with previously published data for* A. adstringens, *which inhibited 95% of mycobacterial activity at the same concentration [10]. Regarding the 5 Clusiaceae extracts, only* C. brasiliense* showed similar potency (82%) as compared with Julianaceae; the other Clusiaceae species inhibited the growth of* M. tuberculosis *H37Rv in the range of 58.3 to 70.3%. Concerning HIV-RT, the Clusiaceae extracts showed inhibition in a range of 27.3 to 67.6%, whereas the Julianaceae extracts inhibited this enzyme in the range of 7.9 to 49.8% (Table 1). Since macrophages are potential targets of* M. tuberculosis* and HIV, in order to assess the cytotoxicity of the extracts, they were tested against macrophages derived from THP1 cells. The extracts inhibited in 9.2–25.5% of the growth of macrophages when tested at 50 *μ*g/mL, suggesting they are innocuous at the tested concentrations (Table 1).

The IC_50_ values were calculated for six species selected for their high activity in both targets. Three of them were Julianaceae (stem bark) and three Clusiaceae (leaves) (Table 2). The six extracts showed potent antimycobacterial activity with IC_50_ in the range 1.8 to 3.8 *μ*g/mL; however, these extracts were less potent inhibiting HIV-RT, since they ranged from 26.2 (*C. brasiliense*) to 97.8 *μ*g/mL (*A. glaucum*). Regarding HIV-RT inhibitory properties of plant extracts, several authors have pointed out that an IC_50_ ≤ 50 *μ*g/mL may be considered potent [23, 24]; however, a similar parameter has not been proposed for* M. tuberculosis*. Assuming the same parameter, the extracts from* C. brasiliense, V. baccifera*, and* V. mexicana* displayed similar potency to both targets, while the extracts from* A. glaucum, A. molle*, and* A*.* simplicifolium* were potent only to* M. tuberculosis* (Table 2).

### 3.2. HPLC Analysis of Extracts of Julianaceae and* C. brasiliense*

The HPLC analysis of the three selected Julianaceae bark extracts showed the following metabolites: masticadienonic acid** 2** and *α*- and/or *β*-hydroxymasticadienonic acids (3 and 4). Under the chromatographic conditions used, quantifying individually the isomers 3 and 4 was not possible due to their similar retention times (Rt = 20.7 and 20.9 min, resp.); therefore, these compounds were quantified as the mixture of acids. Oleanolic acid** 1**, which has been previously reported as a constituent of* A. adstringens* bark, was not detected in the extracts studied (Table 3). The concentration of compounds** 2** and mixture of** 3** and** 4** may not be related to* M. tuberculosis* activity since these three compounds from the most active extracts (IC_50_ < 2.35 *μ*g/mL) were found in high (*A. amplifolia*, male), medium (*A. simplicifolium*), and low (*A. glaucum*, male) content. In addition, the other Julianaceae bark extracts which also showed significant antimycobacterial activity (>84.6% at 50 *μ*g/mL) showed no correlation with the concentrations of the analyzed compounds, as they include the species with the highest concentrations of 2, 3, and 4 (*A. adstringens* from male trees; 14.23% and 10.91%, resp.), but also the species devoid of these compounds (*A. glaucum* female). The above findings suggest that the antimycobacterial active principle in the Julianaceae extracts is not compound** 2**,** 3**, or** 4**. The same can be stated for HIV-RT, since almost all of these extracts, with the exception of* A. simplicifolium*, showed poor activity (Tables 1 and 2).

**Figure 2:**
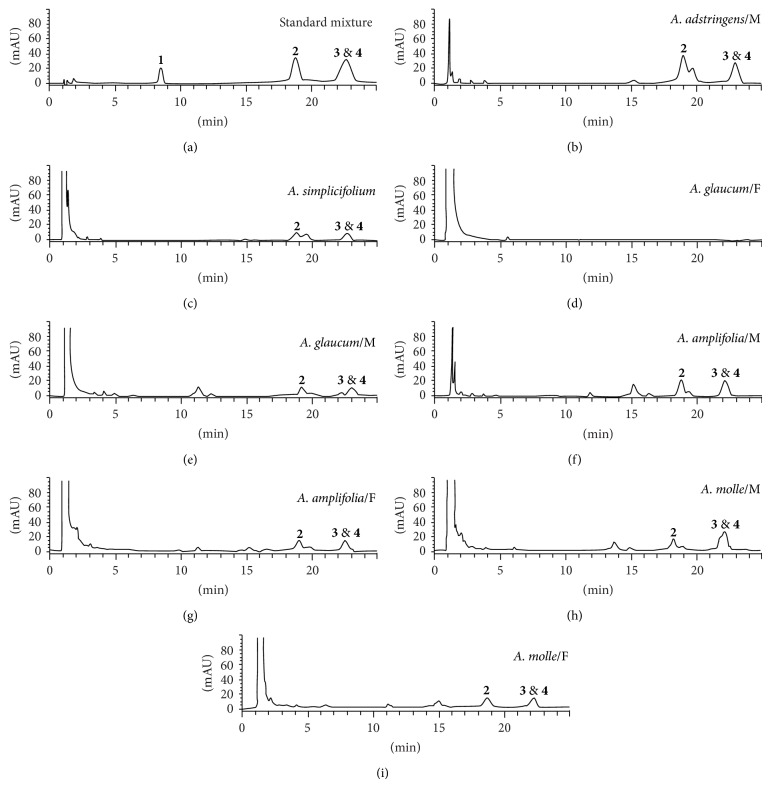
Chromatograms of Julianaceae species extracts and their main triterpenes: oleanolic acid** 1**, masticadienonic acid** 2**, 3*α*-hydroxymasticadienonic acid** 3**, and 3*β*-hydroxymasticadienonic acid** 4**.

With regard to gender and production of secondary metabolites, male specimens showed the higher levels of masticadienonic acid** 2** and 3*α*-hydroxymasticadienonic** 3**, as compared to extracts from female specimens (Figure 2). Our results are opposite to those previously published, in which the accumulation of compounds** 2** and** 3** was higher in female plants [12].

**Figure 3:**
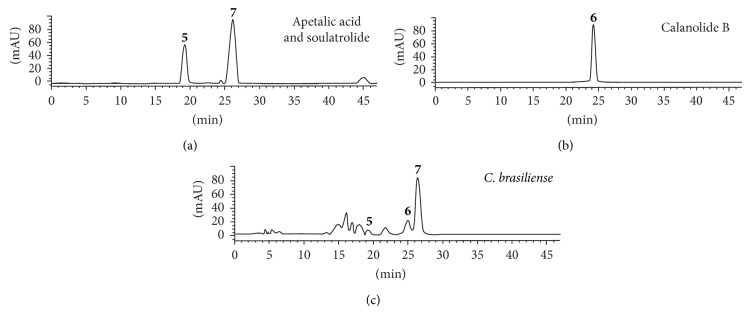
Chromatogram of* C. brasiliense* extract and its compounds: apetalic acid** 5**, calanolide B** 6**, and soulatrolide** 7**.

In the case of Clusiaceae species, only* C. brasiliense* was analyzed by HPLC (Figure 3). Apetalic acid (Rt = 18.64), calanolide B (Rt = 23.34), and soulatrolide (Rt = 25.30) were present in 0.01%, 2.4%, and 6.8%, respectively. Previously, a high antimycobacterial and anti-HIV-RT activity of soulatrolide and calanolide B has been reported [14]. Hence, they can be considered, respectively, as the antimycobacterial and anti-HIV active principles.

## 4. Discussion

HIV infection decreases the number of CD4+ lymphocytes, so it is quite probable that an HIV+ patient can acquire or reactivate tuberculosis disease [25]. During the last 30 years, 24 anti-HIV drugs have been approved by the FDA [9] but any novel anti-TB drug. The rapid spread of multidrug resistance to TB strains remarks that new classes of antimycobacterial compounds are now required [26, 27]. The treatment of patients coinfected with TB/HIV presents also additional challenges, such as intolerance and contraindications for the use of combined drugs and low attachment to medication regime due to the administration of a large number of medications. The highly active retroviral therapy (HAART) for HIV patients involves the administration of a protease inhibitor and two reverse transcriptase inhibitors (1 nonnucleoside + 1 nucleoside type), which represent administration of 20 pills/daily; in addition, monotherapy for TB adds 10 to 12 pills [28]. Moreover, HIV-1 protease inhibitors nullify the effect of the rifampin used as first-line drug for the treatment of TB [26, 27]. In this context, new drugs are needed, if at all possible, active to both targets.

A previous report indicated that one* Amphipterygium* species had a promising activity against TB [10], and our results confirm this finding and extend it to the five species of this genus present in Mexico, which are quite potent against* M. tuberculosis*; however, these extracts showed moderate or poor activity to HIV-RT. No correlation with the content of triterpenoids as masticadienonic acid, 3*α*, and 3*β*-hydromasticadienonic acids was detected for anti-TB or anti-RT activities for the extracts of these species and deserves future investigations in order to identify the active compounds. According to our results* Amphipterygium* species are a source of potent anti-TB extracts with low cytotoxicity to macrophages.

A previous report indicated that the five Clusiaceae species here examined have moderate to high activity against HIV-RT [9], and our results confirm this finding but also show for the first time that they are quite potent to* M. tuberculosis*. In particular,* C. brasiliense* organic extract from the leaves could be suitable for developing a phytodrug due to its content of active molecules to both targets and the calanolides A, B, C, and soulatrolide. Our results also evidence that biodiversity is a useful and valuable source for molecular leads aimed to* M. tuberculosis* and HIV. To date it has been described at least 84 natural compounds active against* M. tuberculosis* [7]. On the other hand, 120 substances, mainly extracted from plants, have been identified with activity* in vitro* against HIV [13]. Only a few of them have been examined for both properties.

## 5. Conclusion

In this study, the high antimycobacterial and moderate anti-HIV-RT activities of Julianaceae bark extracts, especially* Amphipterygium simplicifolium, A. glaucum*, and* A. molle *have been showed. These activities are not related to the triterpenes quantified in this study and suggest that other compounds are the active molecules. Our results provide sustenance to the use of species of Julianaceae plants in Mexican Traditional Medicine in the treatment of tuberculosis. Concerning Clusiaceae, the leaf extracts of the 5 species tested showed good activity against both targets. All the extracts showed low toxicity to human macrophages.* Calophyllum brasiliense* extract may be suitable for developing a phytodrug with dual activity against HIV-1 and* M. tuberculosis *due to its content of the active molecules calanolides and soulatrolide.

## Figures and Tables

**Table 1:** HIV-1 RT and *M. tuberculosis * inhibition by Julianaceae and Clusiaceae extracts^∗^ and their cytotoxicity to THP-1 human cell line.

Species	Location/voucher	Part used/gender	% inhibition of HIV-1 RT	% inhibition of *M. tuberculosis *	% cytotoxicity
Julianaceae					
*Amphipterygium amplifolia *	Jalisco/15637	Stem bark/M	24.8 ± 2.1	90.1 ± 0.6	14.0 ± 1.8
		Leaf/M	18.7 ± 3.2	89.8 ± 0.1	16.3 ± 2.3
	Jalisco/15638	Stem bark/F	36.7 ± 2.8	89.1 ± 0.4	23.3 ± 1.7
		Leaf/F	46.5 ± 4.8	89.7 ± 0.2	25.5 ± 1.9
*Amphipterygium molle *	Jalisco/15639	Stem bark/M	19.3 ± 2.2	89.6 ± 0.5	19.7 ± 1.0
	Jalisco/15640	Stem bark/F	11.3 ± 0.7	88.7 ± 0.7	12.5 ± 1.2
		Leaf/F	49.8 ± 1.8	89.0 ± 0.6	19.5 ± 1.5
*Amphipterygium adstringens *	Jalisco/15641	Stem bark/M	9.2 ± 2.7	88.2 ± 0.1	18.4 ± 1.5
		Leaf/M	6.1 ± 0.7	90.2 ± 0.7	25.1 ± 1.9
*Amphipterygium glaucum *	Michoacán/15644	Stem bark/M	40.0 ± 2.0	86.9 ± 0.5	22.9 ± 1.6
		Leaf/M	48.5 ± 0.7	89.6 ± 0.9	10.9 ± 1.6
	Michoacán/15645	Stem bark/F	21.7 ± 2.1	84.6 ± 1.3	19.7 ± 1.3
*Amphipterygium simplicifolium *	Oaxaca/16125	Stem bark/	44.3 ± 1.2	90.3 ± 0.2	9.2 ± 1.5
		Leaf/	7.9 ± 0.4	90.5 ± 1.0	19.5 ± 0.3
Clusiaceae					
*Vismia mexicana *	Veracruz/134793^§^	Leaf	43.7 ± 0.7	63.5 ± 1.1	10.6 ± 1.4
*Vismia baccifera *	Oaxaca/134792^§^	Leaf	54.0 ± 0.8	70.3 ± 0.5	10.7 ± 1.7
*Clusia guatemalensis *	Oaxaca/134795^§^	Leaf	30.5 ± 2.4	62.1 ± 0.7	17.1 ± 1.3
*Clusia lundellii *	Oaxaca/136723^§^	Leaf	27.3 ± 1.1	58.3 ± 0.4	18.3 ± 1.4
*Calophyllum brasiliense *	Veracruz/15526	Leaf	67.6 ± 1.2	82.8 ± 0.4	23.8 ± 1.1

^∗^CH_2_Cl_2_–MeOH extracts tested at 50 *μ*g/mL. M = male, F = female. Vouchers at IMSSM or FCME^§^.

**Table 2:** IC_50_ of Clusiaceae and Julianaceae extracts.

Species	IC50 ± SEM (*μ*g/mL)	
	*M. tuberculosis* H37rV	VIH-1 RT
*A. glaucum/*M	1.87 ± 1.75	97.83 ± 2.03
*A. molle/*M	2.27 ± 1.52	89.59 ± 1.97
*A. simplicifolium *	2.35 ± 0.97	59.21 ± 1.23
*C. brasiliense *	3.02 ± 1.06	26.24 ± 1.92
*V. baccifera *	3.82 ± 1.19	31.75 ± 1.34
*V. mexicana *	3.64 ± 1.35	36.17 ± 1.53

**Table 3:** Chemical composition (%) of Julianaceae bark extracts.

Extracts	Compounds %		
	1^∗^	2^∗∗^	**3** & **4 **
*A. amplifolia*/M	n/d	8.71	8.77
*A. amplifolia*/F	n/d	4.17	4.42
*A. molle*/M	n/d	4.73	8.53
*A. molle*/F	n/d	3.46	3.35
*A. glaucum*/M	n/d	0.15	3.63
*A. glaucum*/F	n/d	n/d	n/d
*A. adstringens*/M	n/d	14.23	10.91
*A. simplicifolium *	n/d	4.15	3.35

^∗^(RT = 8.06 min), ^∗∗^(RT = 17.29 min), n/d: not detected.
